# Virulence determinants, antimicrobial susceptibility, and molecular profiles of *Erysipelothrix rhusiopathiae* strains isolated from China

**DOI:** 10.1038/emi.2015.69

**Published:** 2015-11-11

**Authors:** Yi Ding, Dongmei Zhu, Jianmin Zhang, Longsheng Yang, Xiangru Wang, Huanchun Chen, Chen Tan

**Affiliations:** 1 State Key Laboratory of Agricultural Microbiology, Huazhong Agricultural University, Wuhan 430070, Hubei Province, China; 2 College of Veterinary Medicine, Huazhong Agricultural University, Wuhan 430070, Hubei Province, China; 3 College of Veterinary Medicine, South China Agricultural University, Guangzhou 510642, Guangdong Province, China; 4 State Key Laboratory of Meat Processing and Quality Control, Jiangsu Yurun Meat Industry Group Co., LTD, Nanjing 210014, Jiangsu Province, China; 5 The Cooperative Innovation Center for Sustainable Pig Production, Huazhong Agricultural University, Wuhan 430070, Hubei Province, China; 6 Key Laboratory of Development of Veterinary Diagnostic Products of Ministry of Agricultural, Huazhong Agricultural University, Wuhan 430070, Hubei Province, China

**Keywords:** epidemiology, *Erysipelothrix rhusiopathiae*, swine

## Abstract

The aim of this study was to understand the epidemiology, serotype, antibiotic sensitivity, and clonal structure of *Erysipelothrix rhusiopathiae* strains in China. Forty-eight strains were collected from seven provinces during the period from 2012 to 2013. Pulse-field electrophoresis identified 32 different patterns which were classified into clonal groups A–D. Most pulsed-field gel electrophoresis (PFGE) patterns were observed in clonal complex A and B, suggesting high diversity of genetic characterization in these two predominant clonal complexes. Antibiotic sensitivity test shows that all the stains were susceptible to ampicillin, erythromycin, and cefotaxime, and resistant to kanamycin, cefazolin, sulfadiazine, and amikacin. Erythromycin and ampicillin are recommended as first-line antibiotics for treatment of *E. rhusiopathiae* in China. The high variation in PFGE pattern among the main clonal groups shows that the *E. rhusiopathiae* in China may originate from different lineages and sources instead of from expansion of a single clonal lineage across different regions.

## Introduction

*Erysipelothrix* are gram-positive, rod-shaped, facultative anaerobic bacteria. Based on phylogenetic relatedness, *Erysipelothrix* spp. can be categorized into *Erysipelothrix rhusiopathiae* (serovars 1a, 1b, 2, 4, 5, 6, 8, 9, 11, 12, 15, 16, 17, 19, 21, and N), *Erysipelothrix tonsilarium* (serovars 2, 7, 10, 14, 20, 22, and 23), *Erysipelothrix* sp. strain 1 (serovar 13), *Erysipelothrix* sp. strain 2 (serovar 18), and *Erysipelothrix inopinata*, a novel species that was recently isolated from sterile-filtered vegetable broth.^1,2,3,4^ These *Erysipelothrix* species are ubiquitous in nature and can cause diseases in a variety of animals including swine, humans, poultry, sheep, cattle, and wild animals. The diseases caused by these species are called erysipelas in animals and erysipeloid in humans.^5^ Humans can be infected with pathogens from the pigs.^6^

Swine erysipelas is caused by *E. rhusiopathiae*, which enters the bloodstream through the tonsils and other lymphoid tissues of the alimentary canal. It is estimated that 30%–50% of healthy pigs carry *E. rhusiopathiae* in the tonsils. The carriers serve as a reservoir for acute erysipelas outbreaks and may not have clinical signs.^7^ The clinical presentation of swine erysipelas are usually manifested as acute septicemia or chronic disease characterized as endocarditis and polyarthritis.^8^

Swine erysipelas appears worldwide and causes economic loss to the swine industry.^9^ Within the Chinese swine industry, the occurrence of erysipelas has significantly decreased since the 1990s due to intensification and improved management of the industry. It only occasionally occurs in small farms. However, since 2010, we have observed an increase in the number of cases of erysipelas submitted to the Huazhong Agricultural University Clinical Microbiology Laboratory. In addition, other research groups have reported similar trends in China.^10^ Whether this increased occurrence of swine erysipelas is due to the development of antimicrobial resistance or extension of a specific lineage is not clear, because information regarding the molecular characteristics and antibiogram of *E. rhusiopathiae* remains limited. To the best of our knowledge, molecular epidemiology of *E. rhusiopathiae* has not been conducted in China.

In the present study, 48 strains of *E. rhusiopathiae* isolated from diseased pigs in China in 2013–2014 were subject to antimicrobial susceptibility testing, virulence gene typing, and pulsed-field gel electrophoresis (PFGE) genotyping to improve our understanding of the virulence traits and molecular profile of *E. rhusiopathiae* in China.

## Materials and methods

The 48 *E. rhusiopathiae* strains were collected from liver, spleen, and blood samples of pigs with a clinical history of acute septicemia by the Clinical Microbiology Laboratory at Huazhong Agricultural University during 2012–2013. These samples were from Southern and Central China, including Zhejiang, Hunan, Hubei, Anhui, Guangdong, Henan, and Jiangsu provinces. Each sample was collected from different pigs and strains with typical morphological characteristics of *E. rhusiopathiae* were picked from each plate. The strains were identified as *E. rhusiopathiae* on the basis of polymerase chain reaction and cellular morphology; growth in gelatin; positive reactions in triple sugar iron agar slants for glucose, lactose, and arabinose fermentation by arginine dihydrolase; and negative reaction for catalase, oxidase, urease, nitrate reduction, mannose, and sucrose fermentation, as described by Wang *et al.*^11^

### Pulsed-field gel electrophoresis (PFGE)

The total DNA of *E. rhusiopathiae* was purified using protocol described previously.^12^ DNA was digested with a mixture of *XbaI*, 0.1% bovine serum albumin, and buffers (Takara Biotechnology, Dalian, China) at 37 °C for 8 h. Digested DNA plugs was loaded in 1% agarose gels and run in an contour-clamped homogeneous electric field (CHEF-DRIII; Bio-Rad, Shanghai, China) for 22 h at 14 °C and 6 V with pulse times from an initial 2.2 s to a final 64 s. The gel was subsequently stained by ethidium bromide for detecting PFGE patterns.

### Antimicrobial susceptibility testing

Susceptibility to ampicillin, erythromycin, cefotaxime, norfloxacin, cefazolin, sulfadiazine, amikacin, polymyxin, tetracycline, doxycycline, lincomycin, and levofloxac in was evaluated by microdilution technique, according to the protocol of the Manual of Antimicrobial Susceptibility Testing described by Stephen *et al.*^13^ Quality control was performed with *Staphylococcus aureus* (ATCC29213) and *Streptococcus pneumonia* (ATCC49619). The breaking point of the minimum inhibitory concentration (MIC) was cited from the Clinical and Laboratory Standards Institute.^14^

### Detection of virulence-associated genes

The presence of capsule synthesis gene (cpsA-C), neuraminidase (nanH.1 and nanH.2), hyaluronidase (hylA-C), surface protective antigen (spa), adhesion, rhusiopathiae surface protein (rspA and rspB), and nine putative virulence genes, including patatin-like phospholipase A and B (locus tag: ERH_0072 and ERH_00334, respectively; GenBank assembly accession: GCA_000270085.1), phospholipase/carboxylesterase family protein A and B (locus tag: ERH_0083 and ERH_0347, respectively; GenBank assembly accession: GCA_000270085.1), lysophospholipase A, B, and C (locus tag: ERH_0148, ERH_1214, and ERH_1433, respectively; GenBank assembly accession: GCA_000270085.1), cardiolipin synthetase, and phospholipase D was investigated using primers described in Supplementary Table S1. Polymerase chain reaction (PCR) was carried out in a 25 μL mixture containing 1 μL DNA, 0.5 μL each primer, 2.5 μL 10× Ex Taq buffer, 2 μL dNTP mixture, 19.25 μL deionized distilled water, and 0.25 μL Ex Taq DNA polymerase (Takara, Dalian, China). Each PCR test was repeated for three times.

### Data analysis

The PFGE patterns were analyzed with BioNumerics software version 6.5 (Applied Maths, Kortrijk, Belgium). A dendrogram was calculated using the unweighted pair group method using arithmetic averages (unweighted pair group method with arithmetic mean (UPGMA)), dice coefficient, and 1.5% optimization with 1.5% band position tolerance. Strains with similarities >70% had eight band differences and were clustered in a clonal complex. Comparisons between different complexes were tested by Fisher's exact test (two-tailed), if a significant result between different complexes was found, comparisons between each complex and all other complexes combined were subsequently tested as well. A *P* value < 0.05 was considered significant.

## Results

### Molecular characterization of *E. rhusiopathiae*

**Figure 1:**
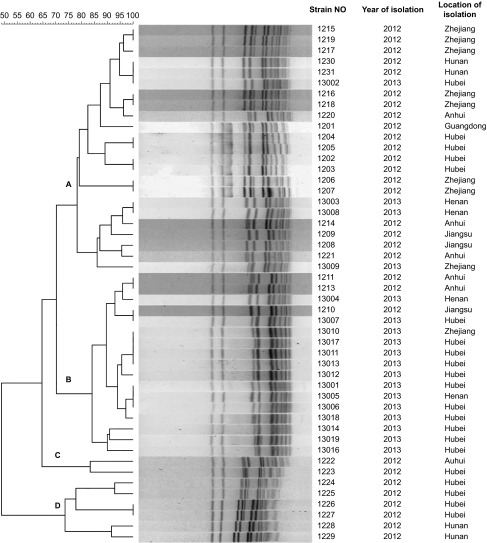
Phylogenetic analysis of PFGE profiles obtained for 48 isolates of *E. rhusiopathiae*. Dendrogram was calculated using the UPGMA method and dice coefficient. Isolates with similarities >70% have eight band differences were considered as closely related and were clustered in a clonal complex, and shown on the left. The strain number, date of isolation, and farm location are indicated on the right.

PFGE resulted in 32 distinct patterns from 48 *E. rhusiopathiae* strains (Figure 1). Dendrogram analysis showed that, at 30% divergence, four clonal groups (A–D) were identified. Twenty-five patterns were observed in clonal complexes A and B, suggesting a high diversity of genetic characterization in these two predominant clonal complexes. Clonal complex A accounted for 48% (23/48) of all strains, with a similarity of 78%–96%. Clonal complex B accounted for 35% (17/48) of strains, with a similarity of 84%–97.5%. Only two strains were classified into clonal group C and showed 83% similarity. Clonal group D included six strains, which showed five different PFGE patterns. Strains with similar PFGE patterns were recovered from different provinces and at different times. Strains 1231 and 13002 shared the same PFGE pattern, but strain 1231 was isolated from Hunan province in 2012 and strain 13002 from Hubei province in 2013. Similarly, strain 1210 isolated from Jiangsu province in 2012 also had the same PFGE pattern as that of strain 13007 isolated from Hubei province in 2013. PFGE pattern of strain 13001, 13005, and 13006 was isolated from samples from Hubei and Henan provinces during the same period.

### Antimicrobial susceptibility

The MICs of 13 antibiotics for the 48 strains are shown in Table 1. All the stains were susceptible to ampicillin, erythromycin, and cefotaxime, whereas all were resistant to kanamycin, cefazolin, sulfadiazine, and amikacin. Differences in antimicrobial susceptibility were detected for gentamicin (47/48 resistant), tetracycline (29/48 resistant), doxycycline (18/48 resistant), lincomycin (37/48 resistant), norfloxacin (37/48 resistant), and levofloxacin (34/48 resistant). With regard to multi-resistance profiles, all strains grouped into 15 resistance phenotypes were resistant to at least five of the ten antimicrobials tested. Thirteen strains were resistant to all six antibiotics mentioned above. Most of them were in clonal complex A (*n* = 8), followed by clonal complexes D (*n* = 3) and B (*n* = 2), and none was found in clonal complex C. There was no difference in antimicrobial susceptibility pattern among the clonal complexes.

### Virulence-associated genes

A total of 21 virulence-associated genes were screened by PCR in this study (Supplementary Table S2). The number of virulence genes detected in *E. rhusiopathiae* ranged from 16 to 21. Strains with all tested virulence genes were evenly distributed among the clonal complexes. Two strains with the fewest virulence genes (*n* = 16) were in clonal complex A, which was the most predominant clonal complex. Capsule polysaccharide synthesis gene (cps-A, B and C), spaA, rspA, nanH. 2, patatin-like phospholipase, lysophospholipase, phospholipase/carboxylesterase, and phospholipase/carboxyl esterase family genes were detected in all *E. rhusiopathiae* strains. Various virulence gene detection rates were observed for the rest of the genes. Among them, phospholipase D and adhesin genes were detected in fewest strains, with detection rates of 63% (30/48) and 77% (37/48), respectively. The virulence gene profiles followed no specific pattern and were independent of the four clonal complexes.

## Discussion

*E. rhusiopathiae* is an opportunistic pathogen that can cause acute septicemia or chronic endocarditis and polyarthritis in pigs.^7^ Diseases caused by *E. rhusiopathiae* generally occur sporadically in China. Nevertheless, we observed an increased frequency of *E. rhusiopathiae* isolation from diseased pigs after 2010. This increasing trend highlights the importance of understanding the molecular epidemiology of *E. rhusiopathiae*. However, there is no previous report about the molecular epidemiology of *E. rhusiopathiae* in China. We therefore genotypically and phenotypically characterized 48 *E. rhusiopathiae* strains from seven provinces of Southern and Central China, to provide a basis for surveillance of this disease in the future.

PFGE is one of the most discriminative typing methods, which provides important insight into population structures of many pathogens, including *E. rhusiopathiae*.^15,16^ Our data showed 32 distinct PFGE patterns from 48 *E. rhusiopathiae* strains. These PFGE patterns were classified into four clonal complexes (A–D). The majority of strains and patterns fell into clonal complexes A and B. The high variation within these predominant clonal complexes indicates that most of the swine erysipelas is not closely related. Therefore, *E. rhusiopathiae* in China may originate from different lineages and sources instead of expansion of a single clonal lineage across different regions. This corresponds to the pathogenic trait of *E. rhusiopathiae* as an opportunistic pathogen that causes sporadic disease in immunocompromised hosts.^13^ However, three PFGE patterns from strain 1231, 13002, 1210, 13007, 13001, 13005 and 13006 were found in different provinces during different times. These clones may spread through trade between these regions or they may possess advantageous traits that facilitate their spread and recurrence.

The antibiotic susceptibility test showed that our strains were all susceptible to ampicillin, erythromycin and cefotaxime, a third-generation cephalosporin. As cefotaxime are not drug intended for use in food animals, ampicillin and erythromycin should be the first choice for the treatment of swine erysipelas in China. Susceptibility of our strains to ampicillin is in line with previous reports in Japan and North America.^12,17,18^ However, resistance to erythromycin was found in 5.8%–6.1% of strains isolated from Japan but not from our collection of *E. rhusiopathiae* strains.^17,18^ The difference in erythromycin resistance in these countries may be due to frequent use of erythromycin for treatment of porcine bacterial respiratory diseases in Japan but not in the swine industry in China.^19^ All *E. rhusiopathiae* strains were resistant to cefazolin, sulfadiazine, amikacin and kanamycin and showed high resistance to gentamicin 98% (47/48), followed by tetracycline 60% (29/48), doxycycline 38% (18/48), lincomycin 77% (37/48), norfloxacin 77% (37/48) and levofloxacin 71% (34/48). Resistance to these antibiotics was in agreement with previous reports by other research groups in Japan, North America and Brazil.^12,20,21^ Only few strains in Brazil was reported to be resistant to fluoquinolones and cefazolin has not been reported before. However, all the *E. rhusiopathiae* strains in this study were resistance to cefazolin and >70% of strains were resistant to norfloxacin and levofloxacin, which are fluoquinolones. The high resistance to these antibiotics indicates over- or misuse of these classes of antibiotics in bacterial infections in swine production in China.

Also, to understand better the virulence potential of our study strains, we characterized 21 virulence-associated genes, including nine putative phospholipase genes and 12 well-characterized and accepted genes: major surface protective antigen (spaA), capsule polysaccharide gene (cpsA-C), rhusiopathiae surface protein (rspA and rspB), hyaluronidase (hylA-C), neuraminidase (nanH.1 and nanH.2), and adhesin, and nine phospholipase genes predicted to be potentially involved in intracellular survival.^14^ The results showed a high detection rate of the above genes in our *E. rhusiopathiae* strains. Eleven genes (cpsA–C, spaA, rspA, rspB, hylA, nanH. 2, patatin-like phospholipase B, lysophospholipase C, and phospholipase/carboxylesterase family genes A and B) were detected in all strains and 30% (15/48) of strains possessed all the virulence genes tested in this study. Phospholipase D and adhesin genes were detected in the fewest strains, with detection rates of 63% (30/48) and 77% (37/48), respectively. The low detection rate of these two genes may have been because they are not essential for the pathogenesis of *E. rhusiopathiae*, or because of the presence of genes with similar function in the genome. Different detection rates were observed for virulence genes hylC, hylA, nanH.1, patatin-like phospholipase A, lysophospholipase A and B, adhesin, and cardiolipinsynthetase; however, there was no correlation between these genes and clonal complexes.

The results of this study could not provide a definitive answer for the current increasing trend of swine erysipelas in China. However, the high variation in phylogenetic characteristics of strains from different regions shows that the increasing trends are caused by strains of various clonal lineages from multiple sources. One explanation for this result is failure to use vaccines. There are three kinds of killed bacterins and two kinds of live-attenuated vaccines for swine erysipelas available in China. However, because of the low occurrence of swine erysipelas after the 1990s, many of the swine farms have not included *E. rhusiopathiae* vaccine in routine regimens. Another explanation may be concurrent virus infection, such as with porcine parvovirus, porcine reproductive and respiratory syndrome virus, and porcine circovirus type 2. These viruses impair the immune system of pigs and either provide a chance for opportunistic pathogens like *E. rhusiopathiae* to infect pigs or reactivate latent infection.

## Figures and Tables

**Table 1:** Distribution of MICs for the 50 *E. rhusiopathiae* strains

	Number of strains with MIC (μg/mL)															
Antibiotics	<0.0625	0.0625	0.125	0.25	0.5	1	2	4	8	16	32	64	128	>128	MIC (μg/mL) on the break point of resistance^a^	Number of resistant strains (%)
Kanamycin														48	64	48 (100%)
Gentamicin							1				2	21	13	11	4	47 (98%)
Ampicillin	33	12	3												0.5	0 (0%)
Erythromycin	13	3	25	7											1	0 (10%)
Cefataxime	8	8	20	9	3										64	0 (100%)
Cefazolin														48	32	48 (100%)
Norfloxacin								1	10	15	13	8	1		16	37 (77%)
Levofloxacin						2	12	23	6	2	2	1			4	34 (71%)
Sulfadiazine														48	512	48 (100%)
Amikacin														48	64	48 (100%)
Tetracycline					3	3	3	3	7	5	9	10	5		16	29 (60%)
Doxycycline			2	5	5	1	1	3	13	6	12				16	18 (38%)
Lincomycin				2	4	5	1	1		2	20	9	3	1	2	37 (77%)

a Resistant strains include intermediate resistant and resistant strains from CLIS.^13^
