# Supplementary material for: Virulence determinants, antimicrobial susceptibility, and molecular profiles of Erysipelothrix rhusiopathiae strains isolated from China
Source: Emerg Microbes Infect. 2015 Nov 11;4(11):e69–. doi: 10.1038/emi.2015.69 (PMC4661428; doi:10.1038/emi.2015.69)
Supplement: Supplementary Information [file emi201569x1.pdf]

Supplementary Table S1 Virulence-associated genes and antimicrobial susceptibility of 48 strains of *E. rhusiopathiae*

	Gene name[a]<br>Predicted function<br>Locus tag<br>Antibiotics	cpsA	cpsB	cpsC	SpaA	rspA	rspB	hylA<br>Hyaluro-nidase<br>ERH-0150	hylB<br>Hyaluro-nidase<br>ERH-0765	hylC<br>Hyaluro-nidase<br>ERH-1210	nanH.1<br>Neura-minidase<br>ERH-0299	Patatin-like phospholipase A<br>ERH-0072	nanH.2<br>Neura-minidase<br>ERH-0761	pldB<br>Lysophospho-lipase A<br>ERH-0148	Phospho-lipase D<br>ERH-0388	Adhesin<br>ERH-1356	Cardiolipin synthetase<br>ERH-0333	Patatin-like phospholipase B<br>ERH-0334	Lysophospho-lipase C<br>ERH-1433
Strain NO.																			
Clonal group A																			
1215	19	√	√	√	√	√	√	√	√	√	√	√	√	√	×	√	×	√	√
1219	20	√	√	√	√	√	√	√	√	√	√	√	√	√	√	×	√	√	√
1217	20	√	√	√	√	√	√	√	√	√	√	√	√	√	√	√	√	√	√
1230	21	√	√	√	√	√	√	√	√	√	√	√	√	√	√	√	√	√	√
1231	20	√	√	√	√	√	√	√	√	√	√	√	√	√	√	×	√	√	√
13002	19	√	√	√	√	√	√	√	√	×	√	√	√	√	×	√	√	√	√
1216	21	√	√	√	√	√	√	√	√	√	√	√	√	√	√	√	√	√	√
1218	16	√	√	√	√	√	√	√	√	×	×	√	√	×	×	×	√	√	√
1220	21	√	√	√	√	√	√	√	√	√	√	√	√	√	√	√	√	√	√
1201	21	√	√	√	√	√	√	√	√	√	√	√	√	√	√	√	√	√	√
1204	20	√	√	√	√	√	√	√	√	√	√	√	√	√	×	√	√	√	√
1205	20	√	√	√	√	√	√	√	√	√	√	√	√	√	×	√	√	√	√
1202	17	√	√	√	√	√	×	√	√	×	√	√	√	×	√	×	√	√	√
1203	21	√	√	√	√	√	√	√	√	√	√	√	√	√	√	√	√	√	√
1206	21	√	√	√	√	√	√	√	√	√	√	√	√	√	√	√	√	√	√
1207	20	√	√	√	√	√	√	√	√	√	√	√	√	×	√	√	√	√	√
13003	20	√	√	√	√	√	√	√	√	×	√	√	√	√	√	√	√	√	√
13008	21	√	√	√	√	√	√	√	√	√	√	√	√	√	√	√	√	√	√
1214	19	√	√	√	√	√	√	√	√	√	√	√	√	√	×	×	√	√	√
1209	16	√	√	√	√	√	√	×	√	√	×	×	√	√	×	×	√	√	√
1208	19	√	√	√	√	√	√	√	√	√	√	×	√	√	√	√	×	√	√
1221	19	√	√	√	√	√	√	√	√	√	√	×	√	√	√	×	√	√	√
13009	21	√	√	√	√	√	√	√	√	√	√	√	√	√	√	√	√	√	√
Clonal group B																			
1211	21	√	√	√	√	√	√	√	√	√	√	√	√	√	√	√	√	√	√
1213	21	√	√	√	√	√	√	√	√	√	√	√	√	√	√	√	√	√	√
13004	19	√	√	√	√	√	√	√	√	√	√	√	√	×	×	√	√	√	√
1210	20	√	√	√	√	√	√	√	√	×	√	√	√	√	√	√	√	√	√
13007	21	√	√	√	√	√	√	√	√	√	√	√	√	√	√	√	√	√	√
13010	20	√	√	√	√	√	√	√	√	×	√	√	√	√	√	√	√	√	√
13017	19	√	√	√	√	√	√	×	√	√	√	√	√	√	×	√	√	√	√
13011	20	√	√	√	√	√	√	√	√	√	√	√	√	√	×	√	√	√	√
13013	21	√	√	√	√	√	√	√	√	√	√	√	√	√	√	√	√	√	√
13012	20	√	√	√	√	√	√	√	√	√	√	√	√	×	√	√	√	√	√
13001	20	√	√	√	√	√	√	√	√	√	×	√	√	√	√	√	√	√	√
13005	19	√	√	√	√	√	√	×	√	×	√	√	√	√	√	√	√	√	√
13006	19	√	√	√	√	√	√	√	√	×	√	√	√	√	×	√	√	√	√
13018	20	√	√	√	√	√	√	√	√	√	√	√	√	√	√	√	√	√	√
13014	21	√	√	√	√	√	√	√	√	√	√	√	√	√	×	√	√	√	√
13019	20	√	√	√	√	√	√	√	√	√	√	√	√	√	×	√	√	√	√
13016	19	√	√	√	√	√	√	√	√	√	√	√	√	√	×	×	√	√	√
Clonal group C																			
1222		√	√	√	√	√	√	√	√	√	√	√	√	√	√	√	√	√	√
1223		√	√	√	√	√	√	√	√	√	√	√	√	√	√	√	√	√	√
Clonal group D																			
1224	20	√	√	√	√	√	√	√	√	√	√	√	√	√	√	×	√	√	√
1225	20	√	√	√	√	√	√	√	√	√	√	√	√	√	√	×	√	√	√
1226	20	√	√	√	√	√	√	×	√	√	√	√	√	√	×	√	√	√	√
1227	19	√	√	√	√	√	√	√	√	√	√	√	√	√	×	×	√	√	√
1228	20	√	√	√	√	√	√	√	√	√	√	√	√	√	×	√	√	√	√
1229	20	√	√	√	√	√	√	√	√	√	√	√	√	√	×	√	√	√	√

Phospholipase/ carboxylesterase B ERH-0347	Lysophos-pholipase B ERH-1214	Phospholipase/ Carboxylesterase A ERH-0083	Norfloxacin MIC (ug/ml)	Tetracycline MIC (ug/ml)	Erythromycin MIC (ug/ml)	Cefazolin MIC (ug/ml)	Cefotaxime MIC (ug/ml)	Docycycline MIC (ug/ml)	Lincomycin MIC (ug/ml)	Gentamicin MIC (ug/ml)	Kanamycin MIC (ug/ml)	Levofloxacin MIC (ug/ml)	Sulfadiazine MIC (ug/ml)	Amikacin MIC (ug/ml)	Ampicillin MIC (ug/ml)
√	√	√	16	64	0.125	512	0.125	16	16	128	512	4	512	512	0.03125
√	√	√	128	128	0.03125	512	0.03125	16	32	64	512	4	512	512	0.015625
√	×	√	32	2	0.125	512	0.0625	0.5	1	64	512	4	512	128	0.03125
√	√	√	64	32	0.03125	512	0.25	32	32	256	512	16	512	512	0.015625
√	√	√	64	64	0.03125	512	0.0625	8	64	128	512	1	512	512	0.015625
√	√	√	64	32	0.03125	512	0.03125	8	64	64	512	2	512	512	0.015625
√	√	√	16	128	0.25	512	0.0625	8	128	128	512	4	512	512	0.0625
√	√	√	32	8	0.125	512	0.0625	16	32	64	512	2	512	512	0.015625
√	√	√	32	32	0.0625	512	0.125	32	32	128	512	4	512	512	0.0625
√	√	√	32	64	0.25	512	0.125	32	32	128	512	4	512	512	0.015625
√	√	√	16	4	0.25	512	0.5	0.5	1	64	512	4	512	512	0.03125
√	√	√	16	1	0.125	512	0.125	0.25	0.5	256	512	2	512	512	0.03125
√	√	√	32	8	0.125	512	0.125	0.5	1	64	512	2	512	512	0.03125
√	√	√	8	2	0.125	512	0.25	0.25	0.5	64	512	1	512	512	0.015625
√	√	√	16	32	0.125	512	0.25	32	128	256	512	8	512	512	0.0625
√	√	√	8	8	0.125	512	0.125	4	32	2	128	4	512	512	0.0625
√	√	√	32	64	0.03125	512	0.0625	4	64	64	512	8	512	512	0.03125
√	√	√	4	16	0.125	512	0.125	8	128	128	512	2	512	512	0.0625
√	√	√	16	128	0.125	512	0.125	32	32	256	512	4	512	512	0.03125
√	√	√	16	64	0.125	512	0.125	32	256	256	512	8	512	512	0.0625
√	√	√	8	64	0.125	512	0.125	32	64	256	512	8	512	512	0.125
√	√	√	8	16	0.125	512	0.25	8	32	64	512	4	512	512	0.03125
√	√	√	32	1	0.03125	512	0.03125	0.25	0.5	64	512	4	512	128	0.015625
√	√	√	8	128	0.25	512	0.125	8	32	128	128	4	512	512	0.0625
√	√	√	32	32	0.125	512	0.125	16	64	256	512	8	512	512	0.0625
√	√	√	16	64	0.03125	512	0.25	8	64	64	512	4	512	512	0.03125
√	√	√	16	128	0.125	512	0.125	32	32	256	512	4	512	512	0.03125
√	√	√	8	1	0.125	512	0.25	0.25	1	128	512	2	512	128	0.015625
√	√	√	32	2	0.125	512	0.125	0.125	0.25	128	512	4	512	512	0.125
√	√	√	8	32	0.25	512	0.03125	8	32	32	512	2	512	512	0.015625
√	√	√	16	16	0.125	512	0.25	0.5	2	128	512	4	512	512	0.03125
√	√	√	8	0.5	0.03125	512	0.03125	0.5	4	64	512	4	512	256	0.03125
√	√	√	32	4	0.125	512	0.125	0.25	0.5	128	512	2	512	256	0.015625
√	√	√	64	32	0.03125	512	0.125	8	32	64	512	2	512	512	0.03125
√	√	√	64	64	0.03125	512	0.0625	4	64	64	512	2	512	512	0.015625
√	√	√	16	64	0.125	512	0.125	8	64	64	512	4	512	512	0.03125
√	√	√	8	64	0.25	512	0.03125	32	64	64	512	4	512	512	0.015625
√	√	√	16	0.5	0.125	512	0.25	1	1	64	512	8	512	256	0.0625
√	√	√	16	32	0.03125	512	0.03125	16	32	32	512	2	512	512	0.015625
√	√	√	8	8	0.125	512	0.03125	2	16	128	512	2	512	512	0.015625
√	√	√	16	8	0.0625	512	0.0625	8	32	64	512	4	512	512	0.015625
√	√	√	32	0.5	0.125	512	0.125	0.125	0.25	64	512	64	512	512	0.015625
√	√	√	32	8	0.125	512	0.5	8	32	64	512	16	512	512	0.0625
√	√	√	16	8	0.0625	512	0.5	8	32	64	512	4	512	512	0.125
√	√	√	64	4	0.125	512	0.0625	16	32	512	512	4	512	512	0.0625
√	√	√	64	16	0.25	512	0.0625	32	32	32	512	4	512	512	0.0625
√	√	√	64	32	0.03125	512	0.25	32	32	256	512	32	512	512	0.015625
√	√	√	32	16	0.03125	512	0.125	32	32	128	512	32	512	512	0.015625

[a] √: gene is detected; ×: gene is not detected

Supplementary Table S2 Primer sequences used to amplify virulence associated genes

Target Gene/Locus tag	Primer sequence	Annealing (℃)	Size of product (bp)	reference
SpaA	ACACCTATTTCCGAAAGTAA	51	1800	This study
	CTATTTTAAACTTCCATCGTT			
rspA	ATATTTGGATTCATGGGAGGG	53	6024	This study
	TACACGCATGTAGGTTTTGTT			
rspB	AAGTTCACAAGCAAAGGAGA	53	2402	This study
	AGCTTCTATTCTTTTTCCTCAC			
cpsA	GAGTGGTATTTTATGGTCATT	52	1106	This study
	GCTTACTATTTTATCGTGTATC			
cpsB	CATTATACCGTTGCACACGC	54	1156	This study
	AGTACAGTGTGACCAAGATG			
cpsC	GGCTAAGTTAGTCGATCTTT	52	1256	This study
	CATCACTTGATTCTAAGCGA			
ERH-0150	CTCCATTATCGCTTACAACAAC	52	4037	Ogawa et al[a]
	AAAACGGCGCGAATTTA			
ERH-0765	TCAAACAGTCAATACCAAACACCGTTGTCTCGGGTTTCAC	54	1030	Ogawa et al
ERH-1210	TTGTTTCCAAAAATCTTCAATGTCCCTTCACATTATAATAATAGGAA	55	3978	Ogawa et al
ERH-0299	CCCTGAGATGATGCTTGAAA	54	4621	Ogawa et al
	CGGTAAAGCAGTAACCTTCAC			
ERH-0072	ATGAATAATAGAATTTGTATCATATAGACTTCGCAGTATCAATATGACGATAT	53	1181	Ogawa et al
ERH-0761	CGACTCTCATTTTTAAAATCATCT	53	1561	Ogawa et al
	CTGAAATTGTATCACAAACTGTGA			
ERH-0148	AGTTGGTGGTGCATTCACTTA	53	1021	Ogawa et al
	CAAGATGTGTTTCGATATAATCAA			
ERH-0388	TATCTATATTTTTGACTTCTTAATACTCG	55	961	Ogawa et al
	GTCGGAGCGGGATCTGT			
ERH-1356	TCCATTGACAAAAAAGAAGAAC	53	1141	Ogawa et al
	GATGATGGACCTGAGCATAAG			
ERH-0333	TACTTCAATTAAATTATCATCTCGC	54	1985	Ogawa et al
	GGGCGAATCCCTACATTG			
ERH-0334	ATGATGTAATGCATACCATTACAG	54	1111	Ogawa et al
	GCACGCTATAATAAGCACTCA			
ERH-1433	TTAGCATCATTTTTTAGATGATTT	51	1111	Ogawa et al
	CGTCTCCTATTTTCACGACTT			
ERH-0347	AGCCCTTCTATCCAGTTAGC	54	783	Ogawa et al
	TTATAGATACGATTTGCGTAGGT			
ERH-1214	GTTGAATCGTGTCGTTTATCC	54	1049	Ogawa et al
	AAAGCTGTATCTAATGCACTCTC			
ERH-0083	AAGAAATGCGTAAGCGATT	52	772	Ogawa et al
	GTTCCGTGTTGTTATCCAAG			

[a] Ogawa Y, Ooka T, Shi F, Ogura Y, Nakayama K, Hayashi T, et al. The genome of *Erysipelothrixrhusiopathiae*, the causative agent of swine erysipelas, reveals new insights into evolution of firmicutes and the organism's intracellular adaptations. J Bacteriol. 2011; 193: 2959–71.
